# Affinity of anti-spike antibodies to three major SARS-CoV-2 variants in recipients of three major vaccines

**DOI:** 10.1038/s43856-022-00174-9

**Published:** 2022-08-25

**Authors:** Patrick J. Macdonald, Jeffrey M. Schaub, Qiaoqiao Ruan, Carroll L. Williams, John C. Prostko, Sergey Y. Tetin

**Affiliations:** Applied Research and Technology, Abbott Diagnostics Division, Abbott Laboratories, Abbott Park, IL USA

**Keywords:** Antibodies, Vaccines

## Abstract

**Background:**

Measuring anti-viral antibody affinity in blood plasma or serum is a rational quantitative approach to assess humoral immune response and acquired protection. Three common vaccines against SARS-CoV-2—Comirnaty developed by Pfizer/BioNTech, Spikevax developed by Moderna/NIAID, and Jcovden (previously Janssen COVID-19 Vaccine) developed by Johnson & Johnson/Janssen (J&J)—induce antibodies to a variety of immunogenic epitopes including the epitopes located in the ACE2 receptor-binding domain (RBD) of the spike protein. Blocking RBD with antibodies interferes with the binding of the virus to ACE2 thus protecting against infection.

**Methods:**

We perform measurements in the serum of the recipients of Pfizer, Moderna, and J&J vaccines, and we compare the apparent affinities of vaccine-induced antibodies against the RBD of the ancestral SARS-CoV-2 virus and the Delta and Omicron variants. We use our recently published method to determine the apparent affinity of anti-spike protein antibodies directly in human serum. This involves probing antibody-antigen equilibria with a small number of antigen-coated magnetic microparticles and imaging them on a fluorescence microscope.

**Results:**

Recipients of two-dose Pfizer and Moderna vaccines, as well as recipients of the single-dose J&J vaccine, develop high-affinity antibodies toward RBD derived from ancestral SARS-CoV-2. Affinities of these antibodies to Delta-RBD are approximately 10 times weaker, and even more drastically reduced (∼1000-fold) toward Omicron-RBD.

**Conclusions:**

Vaccine-induced antibodies against ancestral SARS-CoV-2 RBD demonstrate ~10-fold and ~1000-fold weaker affinities toward Delta- and Omicron-RBD, respectively. Our approach offers a direct means for evaluating vaccine-induced adaptive immunity and can be helpful in designing or updating vaccines.

## Plain language summary

Vaccination results in the production of proteins called antibodies that can bind viruses. The strength (affinity) of the binding between an antibody and a virus gives an indication of how well vaccination can prevent infection by the virus. Here, we compare the affinity of antibodies towards different types (variants) of SARS-CoV-2 viral protein in the serum of individuals who have been vaccinated with vaccines produced by Pfizer (Comirnaty), Moderna (Spikevax), and Johnson & Johnson (Jcovden). We discovered that all three vaccines generated antibodies that strongly bind the original SARS-CoV-2 virus, but the binding of the Delta variant was ten-times weaker. Moreover, the binding of the antibodies to the Omicron variant was more than a thousand-times weaker. Our findings explain why current vaccines are less efficient at preventing infection by the Delta and Omicron SARS-CoV-2 variants.

## Abstract (web summary)

Macdonald, Schaub et al. measure apparent antibody affinities to the ACE-2 receptor binding domain of the SARS-CoV-2 spike protein in recipients of the Pfizer, Moderna and J&J vaccines. All vaccinated individuals develop high-affinity antibodies, however the affinities are weaker to Delta and Omicron variants compared to ancestral virus.

## Introduction

Measuring anti-viral antibody affinity in blood plasma or serum is a rational, quantitative approach to assessing humoral immune responses and adaptive immunity. Three common vaccines against SARS-CoV-2, developed by Pfizer/BioNTech, Moderna/NIAID, and Johnson & Johnson/Janssen (J&J), induce antibodies to a variety of immunogenic epitopes, including the epitopes located in the receptor-binding domain (RBD) of the spike protein. Spike RBD mediates the binding with membrane-bound angiotensin-converting enzyme 2, commonly known as the ACE2 receptor, which is the precursor for the entry of the SARS-CoV-2 virus into human cells. ACE2 is located on the surface of various human epithelial cells and serves as the anchoring point for the virus to breach the membrane^1–3^. Antibodies directed toward the receptor-binding domain of viral spike protein S1 can interfere with the binding of the virus to host cell ACE2 and thus prevent infection^4–6^. Additionally, studies have shown that nearly 90% of neutralizing antibody activity in SARS-CoV-2 patients is related to spike RBD^7,8^. Therefore, assessing the affinities of anti-RBD antibodies, generated in response to vaccination against SARS-CoV-2, can serve as a quantitative measure of the humoral immune response and acquired anti-viral protection against ancestral SARS-CoV-2^9^ and its Delta and Omicron variants^10,11^.

It was recently found that the neutralizing effect of monoclonal antibodies developed against ancestral SARS-CoV-2 is not as effective against the mutated variants^12,13^. Reductions in neutralization efficacy have also been observed for vaccine-induced antibodies^14,15^. However, the reported neutralizing antibody titers encompass two primary parameters that drive the RBD and anti-RBD binding reaction: antibody affinity and antibody concentration, which should be quantified independently.

In our previous publication^16^, we presented a simple imaging-based method for measuring antibody affinities independently of concentration. This was done directly in human blood serum or plasma without purification or labeling and used to follow the development of the humoral immune response in convalescent COVID-19 patients. We also showed that high antibody affinities can be achieved faster by vaccination than from infection.

In this work, we extend this method to directly compare the antibody affinities to RBD from the ancestral SARS-CoV-2 strain and its Delta and Omicron variants. We show that recipients of Pfizer, Johnson & Johnson, and Moderna vaccines develop high-affinity antibodies to RBD derived from the ancestral SARS-CoV-2. The affinity of these antibodies is ~10-times weaker to the Delta-RBD, and ∼1000-fold weaker to the Omicron-RBD.

## Methods

Serum was highly diluted to bring anti-spike antibody to similarly low levels for all samples, on the order of tens of picomolar (∼5 µg/L). Maintaining the antibody concentration well below the expected dissociation constant (K_D_) makes the measured affinity independent of antibody concentration. Twenty-four aliquots of diluted serum (patient antibody) were incubated with serially titrated RBD antigen to achieve equilibrium. Subsequently, a small number of magnetic microparticles, coated with wild-type RBD, were added to the reaction solutions to probe the amount of free antibody (not bound to antigen) without noticeably perturbing the equilibria. The microparticles carrying captured antibodies were washed, reacted with fluorescently labeled anti-human IgG conjugate, washed again, and imaged on a fluorescence microscope. Using the zero-RBD titration point values as the maximum fluorescence intensity, it is possible to quantify the fraction of antibody prevented from microparticle capture due to the prior formation of antibody-RBD complex. Plotting the fraction of blocked antibody (% inhibition) as a function of RBD concentration generates a binding curve. Fitting the curve yields an apparent affinity of the polyclonal antibodies.

### Fluorescence imaging

Fluorescently labeled magnetic microparticles were imaged in optical-bottom 96-well plates on an IX83 inverted microscope (Olympus, Tokyo, Japan). Epifluorescence excitation was produced using an X-Cite LED illumination system (Excelitas, Wheeling, IL). An automated stage was employed to obtain 9 acquisitions per well using a pco.panda camera (PCO, Kelheim, Germany) and a 20x air objective (UPlanXApo, NA = 0.80, Olympus). Each acquisition consisted of a brightfield image to locate the microparticles and a fluorescent image (Cy3 filter cube, Edmond Optics, Barrington, NJ) to record the fluorescence intensity. Imaging analysis, including large fluorescent aggregate removal (e.g., dust/hair), calculation of the total microparticle pixel area and total microparticle fluorescence, was performed in Metamorph Advanced software (Molecular Devices, San Jose, CA), as previously described^17^.

### Reagents

ARCHITECT wash buffer (phosphate buffer containing detergent, Abbott Laboratories, Abbott Park, IL) served as the primary diluent for all experiments. Three variants of recombinant receptor-binding domain (RBD) of the S1 subunit spike protein served as antigens. CHO-cell expressed, wild-type RBD (residues 319–541) was produced in-house. SARS-CoV-2 Delta (B.1.617.2) and Omicron (B.1.1.529) variant RBD proteins were purchased from GenScript (Piscataway, NJ). The Delta-RBD construct consists of residues 319–591 and includes the L452R and T478K mutations, and the Omicron-RBD (residues 319–541) has 15 mutations. Magnetic microparticles (4.7 µm, Agilent, Santa Clara, CA), coated with the same recombinant WT-RBD, were used to capture anti-spike antibody in serum samples. Captured antibodies were detected using Cy3-labeled donkey anti-human IgG antibody conjugate (Jackson ImmunoResearch, West Grove, PA).

### Determining serum dilutions

To determine antibody affinities which are independent of antibody concentrations, the antibody concentration in the titration experiments must be close to or below the binding affinity dissociation constant (on the close order of 1 nM). To provide an initial estimate of the anti-spike antibody levels in the patient serum samples, a SARS-CoV-2 IgG II Quant ARCHITECT assay was performed using a commercially available assay kit, according to the package instructions (link to the package insert: https://www.corelaboratory.abbott/us/en/offerings/segments/infectious-disease). Subsequently, this data was used to create the initial estimated serum dilutions. A single aliquot of diluted serum from each patient was run through the experimental protocol below to determine the fluorescence intensity level, which is proportional to the antibody level. An intensity signal of ∼2000 counts was targeted, being the optimal balance between minimizing antibody concentration and maintaining a clearly detectable signal. The results of these single patient aliquot experiments were used to further refine individual patient dilution factors to bring all samples to approximately the same low antibody level.

### Identifying presumably pre-infected individuals

As mentioned above, we employed a SARS-CoV-2 IgG II Quant ARCHITECT assay to quantitate IgG antibodies against the spike receptor-binding domain (RBD) of SARS-CoV-2. These assays were performed on the post-vaccination serum samples as described above, but also on the pre-vaccination samples associated with each individual. The assay cut-off value for detecting the presence of anti-RBD IgG is 50 AU/mL, as per the assay insert. Pre-vaccination sample IgG levels above this value are presumed to indicate that the individual experienced a prior SARS-CoV-2 infection.

### Apparent affinity titration experiments

Patient sample was diluted in ARCHITECT wash buffer to achieve low antibody concentrations (∼60 pM), well below the expected dissociation constant of the target binding reactions, and was aliquoted into 24 wells of two 96-well plates. Different SARS-CoV-2 variants of RBD were titrated into the patient sample wells starting at 5 µM RBD, next at 1 µM RBD and then in two-fold serial dilutions down to 2 pM, plus additional wells containing no RBD (buffer only) as zero titration point controls. All assay steps were carried out on a KingFisher magnetic microparticle plate processor (Thermo Fisher Scientific, Waltham, MA) maintained at 37 °C. The patient aliquots with titrated RBD were incubated for 30 min to equilibrate the antibody-antigen binding reaction. Subsequently, 2 µL of RBD-coated microparticles (0.1% solids) were added to the reactions and incubated for 5 min to sample the amount of patient antibody not already bound to RBD. The microparticles with captured antibody were washed with ARCHITECT wash buffer, incubated for 10 min with 80 µL of the Cy3-labeled anti-human conjugate (3.6 nM), washed again, and imaged on a fluorescence microscope. Two pseudo-data points at extremely high concentrations (100% inhibition at 1 mM and 10 mM RBD, see Apparent affinity calculations below) were appended to the experimental data, and the resulting binding curves were fit to a standard four-parameter logistic model in Origin 2016 (OriginLab, Northampton, MA). The recovered RBD concentration at 50% inhibition is an apparent dissociation constant of the binding reaction since the patient produces a polyclonal antibody response. The inverse of this value is the apparent affinity of the polyclonal antibodies. Considering the probable steric hindrance effect caused by the relative sizes of the RBD antigen (~30 kDa) and the antibodies interacting in solution, we do not expect a large fraction of RBD molecules to bind to more than one antibody. As such, the apparent affinity should be a measure of largely one-on-one interactions between the antigen and antibody.

### Apparent affinity calculations

The highest RBD concentration employed in the titration experiments for all three variants was 5 µM. As the observed antibody affinities to Omicron-RBD are quite weak, this means that in most cases the Omicron binding curves only barely reach 50% inhibition and none achieve 100% inhibition. It is impractical to use RBD concentrations that are much higher since protein aggregation and nonspecific background noise begins to compromise the results. As such, the data did not justify extrapolating a 50% binding point beyond 5 µM (or 5000 nM). Thus, any Omicron affinities weaker than 0.0002 × 10^9 ^M^−1^ (i.e., 1/5000 nM) were fixed to 0.0002, nor was any value lower than 0.0002 used in calculating the relative affinities. Although, for the purposes of this work, 0.0002 represents a lower bound, many of those samples likely have affinities to Omicron-RBD that are still weaker. It is also important to note that, with regards to the weaker affinities to Delta- and especially to Omicron-RBD, we have made a further assumption in fitting the data. WT-RBD curves saturate at a high WT-RBD concentration which establishes 100% inhibition (i.e., all of the patient’s antibody was bound to RBD in solution) and defines the top of the binding curve. At this saturation point, the measured fluorescence signal comes from nonspecific binding of other, non-SARS-CoV-2 antibodies from that patient. As this value is independent of the RBD variant used to bind the anti-spike antibodies, it can be taken from the WT-RBD experiments and applied to those of the Delta- and Omicron-RBD curves, pinning the endpoint of the binding curve fits to the same value for all three variants. In practice, this was accomplished by appending pseudo-data points (non-experimental) for 100% inhibition at extreme concentration values, 1 mM and 10 mM, for all three variants.

### SARS-CoV-2 patient samples

Serum samples from random, unidentified individuals who received either two doses of the Pfizer/BioNTech vaccine, one dose of the Johnson & Johnson/Janssen vaccine, or two doses of the Moderna vaccine were purchased from Access Biologicals, LLC (Vista, CA). Additional Pfizer and Johnson & Johnson vaccine panels were purchased from Precision for Medicine (Norton, MA). For each vaccine panel, the serum samples used in this study were drawn approximately 35–60 days after the individual received their first dose of vaccine. Total numbers of patients measured in this study were as follows: Pfizer: 26 individuals, Moderna: 29 individuals, and J&J: 19 individuals. Six additional patients were not included in this study as they were deemed not measurable due to very low antibodies or very high background (Pfizer: 3, Moderna: 1, J&J: 2).

### Ethics

Patient serum samples used for this study were purchased from Access Biologicals, LLC and Precision for Medicine and collected with informed consent. The samples purchased from Access Biologicals, LLC were collected under an IRB protocol approved by Ballad Health System IRB (IRB #00003204). The samples purchased from Precision for Medicine were collected under an IRB protocol approved by IntegReview IRB, LLC (IRB00008463).

### Statistics and reproducibility

Fluorescence signals and apparent affinity values are reported as the mean ± ${SD}/{mean}.$ Fluorescence signal uncertainties were obtained from multiple images of the same sample (*n* = 9), as described above. Box plots display interquartile range (Q1–Q3), median, and mean, along with whiskers to min/max values. One-way ANOVA was performed to determine if there was a statistically significant difference in affinity among the three vaccinated groups—Pfizer, J&J, or Moderna. Two-way ANOVA was performed to determine if there was a statistically significant difference in relative affinity across the three vaccine groups and two variants. A One-way ANOVA was performed to determine if there was a statistically significant difference in anti-spike antibody concentration levels among the three vaccinated groups. If there was a significant difference among the three groups, a pairwise comparison was performed using Tukey’s Honest Significant Differences (HSD) Test to determine which vaccinated groups were significantly different. Tukey’s HSD test was performed with a 95% confidence level. Analyses performed in JUMP 16.0 (SAS, Cary, NC).

### Reporting summary

Further information on research design is available in the Nature Research Reporting Summary linked to this article.

## Results

As we showed in a previous publication^16^, we developed a method to determine the apparent affinity of anti-viral antibody to its antigen by performing binding titrations directly in patient serum. It is important to underscore that the apparent affinity value obtained by this method is *independent* of antibody concentration and is a measure of the functional binding strength of the polyclonal antibodies. Apparent affinity is a quantifiable parameter and is determined as the midpoint of the binding titration series. In fact, it represents a combination of variable stoichiometries, affinities, and avidities^18^. We used apparent affinity to study the anti-SARS-CoV-2 immune response by following the development of high-affinity antibodies in convalescent patients and vaccinated individuals. We also showed how changes in patient antibody affinity can bias quantitative COVID-19 antibody assays.

With the continuation of the COVID-19 pandemic, i.e., the onset of the Delta and Omicron variants, and with growing evidence of vaccine escape, we decided to quantitate the affinities of vaccine-induced anti-spike antibodies to Delta- and Omicron-RBD, as compared to the original or wild-type (WT) strain of SARS-CoV-2. To this end, we acquired serum samples from individuals who received two-dose Pfizer-BioNTech or Moderna/NIAID vaccines, or the single-dose Johnson & Johnson/Janssen (J&J) vaccine. The blood was drawn ~7–9 weeks after the initial administration of vaccine.

**Fig. 1:**
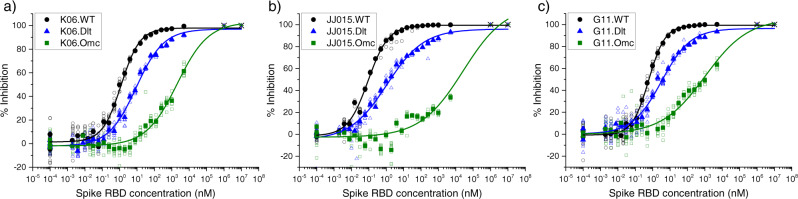
Representative antibody binding curves toward SARS-CoV-2 RBD variants. Binding curves for interactions between patient antibodies and RBD from the WT (black circles), Delta (blue triangles), and Omicron (green squares) variants of SARS-CoV-2 are shown for three representative, single individuals who received either (**a**) Pfizer, (**b**) J&J, or (**c**) Moderna vaccination. For each representative individual, the binding curves for Delta- and Omicron-RBD show clear shifts to the right, indicating weaker affinities of the individual’s anti-spike antibody. Fixed, pseudo-100%-inhibition points for Delta- and Omicron-RBD (“*” symbols) were appended to all data sets at 1 mM and 10 mM prior to the fit (see Methods). Solid symbols: average, open symbols: nine technical replicates.

We applied our antibody affinity determination method to these vaccine panels with respect to each of the three RBD variants. Figure 1 shows binding curve data from three representative individuals, each vaccinated by one of the three primary vaccine types—(a) Pfizer, (b) J&J, and (c) Moderna. Each plot displays the data and logistic model fits for the binding reactions between the individual’s antibody and either WT-, Delta-, or Omicron-RBD. All examples show a similar trend in decreasing affinity for Delta- and Omicron-RBD compared to WT-RBD. Not surprisingly, the vaccine-induced patient antibodies show high-affinity binding to the WT-RBD since the all vaccines are based on WT spike protein^19^ and its highly immunogenic receptor binding domain^7,8^. Delta-RBD contains two mutations and Omicron-RBD has fifteen mutations compared to WT. The shifts of the binding curves to the right indicate that the patient antibodies have weaker affinities toward Delta-RBD and, especially, Omicron-RBD. Higher concentrations of Delta- and Omicron-RBD were required to achieve comparable binding fractions. The complete results from three vaccine panels (Pfizer: 26 individuals, Moderna: 29 individuals, and J&J: 19 individuals) determining apparent affinities toward each of the three RBD variants are shown in Supplementary Tables S1–S3. Supplementary Tables S4–S6 provide detailed information about the vaccine panels. Binding curves for all studied samples are presented in Supplementary Figs. S1–S3.

**Fig. 2:**
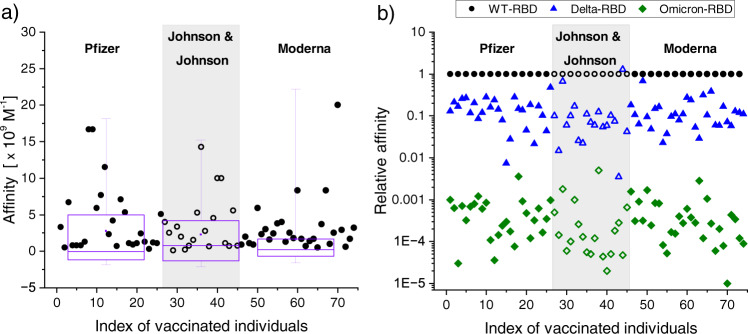
Anti–SARS-CoV-2 WT-RBD affinities and relative affinities toward variants. **a** All affinities of patient anti-spike antibodies to WT-RBD are simply plotted against an index of the vaccinated individuals, grouped by vaccine make. All three vaccines induce similar, high-affinity antibodies toward WT-RBD. There is no significant difference in affinity among the three vaccine groups (F-test *p*-value = 0.7051). **b** Relative antibody affinities of individual patients to RBD variants (Delta, Omicron) as compared to WT-RBD (normalized to 1). As before, these relative affinities are plotted against patient index. The results show a remarkably consistent trend (no significant difference among the three vaccine groups, F-test *p*-value = 0.9018). All vaccine-induced antibodies have approximately a 10-fold weaker affinity toward Delta-RBD and a 1000-fold weaker affinity toward Omicron-RBD. The differences in relative affinities toward the variants are statistically significant (F-test *p*-value = 0.0000). The number of samples tested was 26, 19, and 29 for Pfizer, Johnson & Johnson, and Moderna, respectively.

The anti–WT-RBD antibody affinities induced by the different vaccines are displayed in Fig. 2a, simply plotted as a function of patient index, and grouped by vaccine make. Each vaccine induces high-affinity antibodies with expected patient-to-patient variability, all clustering in the same range, 1 × 10^9 ^M^−1^ – 5 × 10^9 ^M^−1^. For easier comparison, we generated a normalized plot of the affinities to Delta- and Omicron-RBD with respect to that of WT-RBD for each immunized individual (Fig. 2b). The *x*-axis is patient index and the *y*-axis is presented in log scale since relative affinity covers several orders of magnitude. The log scale somewhat suppresses the appearance of patient-to-patient variability which is substantial due to wide variations in immune responses. Nevertheless, the overall pattern is clear. Across all three vaccinated groups, the antibody affinities to Delta-RBD are approximately 10-fold weaker, and the affinities to Omicron-RBD are more than 1000-fold weaker, compared to WT-RBD.

## Discussion

**Fig. 3:**
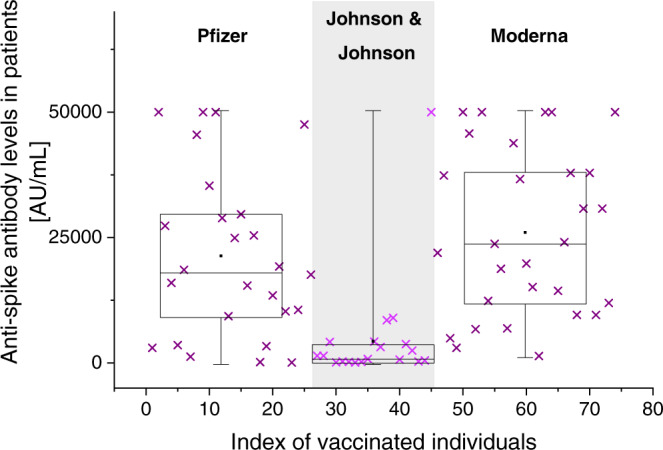
Anti–SARS-CoV-2 antibody concentration levels. Anti-spike IgG antibody levels in patient samples were measured using Abbott’s SARS-CoV-2 IgG II Quant ARCHITECT assay. Comparing these antibody concentrations, the one-dose J&J vaccine induces lower antibody levels than the two-dose Pfizer and Moderna vaccines. There is no significant difference in mean concentrations between individuals vaccinated with Pfizer vs Moderna (the Tukey HSD test *p*-value = 0.5108). However, there is a significant difference in antibody concentration levels across the three groups as determined by ANOVA (F-test *p*-value = 0.0001), and pairwise Tukey HSD (Pfizer vs J&J: *p*-value = 0.0019; Moderna vs J&J: *p*-value = 0.0000). The number of samples tested was 26, 19, and 29 for Pfizer, Johnson & Johnson, and Moderna, respectively.

The affinities of circulated antibodies reflect the overall status of the adaptive immune system and thus serve as a quantitative measure of a specific humoral immune response. Our binding titration data clearly indicate that all three vaccines induce anti-spike antibodies of similar *affinities*. However, as expected, the one-dose (J&J) and the two-dose (Pfizer, Moderna) vaccines do not induce equal *levels* of anti-RBD IgG antibody (Fig. 3) when measured at comparable times following the initial vaccine administration. Indeed, having the second vaccine injection boosts antibody production. From a mechanistic perspective of the humoral immune response, the processes of B-cell clone selection and antibody affinity maturation^20,21^ are triggered by the first exposure to antigen. Additional inoculations enhance clone proliferation and antibody production, but do not appear to drastically affect the basic dynamics of the adaptive immune response. Affinity and concentration function together to determine overall immunity, but they remain independent parameters. Low-affinity antibodies at a sufficiently high concentration are still likely to have a beneficial effect, but a high concentration of high-affinity antibodies is obviously ideal. Being able to distinguish the contributions of the affinity and concentration is important for understanding how to combat any loss in vaccine efficacy.

Measuring binding parameters in solution is standard biophysical practice as it avoids the inhomogeneous binding and surface effect artifacts often observed in other measurement techniques such as ELISA, biolayer interferometry (BLI), and surface plasmon resonance (SPR)^22,23^. The binding of antibodies to surfaces is restricted by diffusion, a phenomenon known as the mass transport limitation. Additionally, antibody-antigen dissociation from surfaces may be impaired by rebinding to the surface which causes artificially decreased dissociation rates. Such limitations are well-known and have been previously discussed^24^.

Recent neutralization publications have also demonstrated decreased vaccine efficacy against the Delta and Omicron variants. However, the neutralization efficiency includes a convolution of both antibody affinity *and* concentration, which precludes quantification of either property. In our study, we diluted each antibody sample below the expected dissociation constant to ensure that the obtained affinity was *independent* of concentration^16^. We then probed the titration reaction with a minute number of microparticles to minimize perturbation of the solution-phase equilibrium. Our data indicate greater apparent affinity decreases to the Delta and Omicron variants in comparison to the abovementioned viral neutralization studies. This is consistent with earlier studies showing that virus neutralization titers correlate with—but are less sensitive to—changes in affinity^18^.

Based on clearly measurable levels of anti-SARS-CoV-2 antibody in serum samples prior to vaccination, some individuals were identified as likely having had a WT-SARS-CoV-2 infection before they were vaccinated. This leads to higher affinities toward WT-RBD, compared to the general population of vaccinated individuals (Supplementary Fig. S4a), which is consistent with observations from our previous work^16^. In fact, most of the individuals showing affinities greater than 4 × 10^9^ M^−1^ in Fig. 2a fall in this category. In general, these individuals also showed higher absolute affinity values toward Delta- and Omicron-RBD, but the relative affinity decreases toward the variants (~10-fold and ~1000-fold) remain consistent (Supplementary Fig. S4b).

**Fig. 4:**
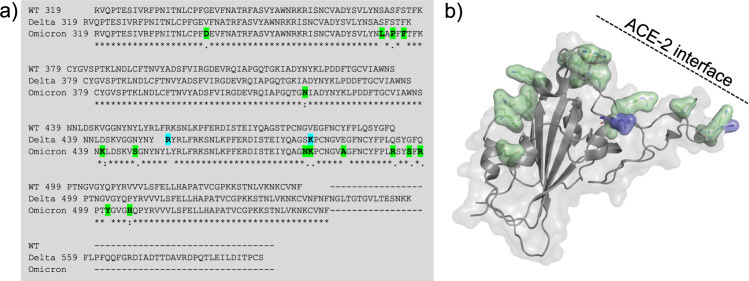
Sequence alignment and structures of SARS-CoV-2 RBD variants. **a** The sequence alignment chart of the RBD constructs used in this study, showing mutations noted in blue (Delta) and green (Omicron). **b** Structural surface and cartoon representation of WT SARS-CoV-2 RBD (grey, PDB: 6LZG), with color-coded surface mutations for Delta- (blue, PDB: 7WBQ) and Omicron-RBD (green, PDB: 7WBP)^28,29^. Note that most mutations cluster on the ACE2 binding interface.

It is well known that a protein region which is immunogenic (induces an immune response) may consist of several antigen epitopes (footprints of antibodies on the antigen). There are continuous (linear) and discontinuous (conformational) antigenic protein epitopes which may be discrete or overlapping^25–27^. Considering the large shifts in affinity toward WT-, Delta-, and Omicron-RBD, as well as the consistency observed across the three vaccine makes, we suggest the existence of a common antigenic epitope region targeted by anti-RBD antibodies. Further speculation, based on the relative sizes of the affinity shifts, would place one of the Delta mutations within this epitope and at least one more of the Omicron mutations in the same region. A simple scan of the aligned RBD sequences (Fig. 4a) and three-dimensional structures^28,29^ (Fig. 4b) reveals that several amino acids in the loop centered around residue 478 form an exposed surface that includes both Delta and Omicron mutations. While outside the scope of our work, this would appear to be a good starting point for an epitope mapping project.

Overall, this study clearly shows significant and consistent decreases in antibody affinities toward the Delta and Omicron RBDs. While not unexpected, given reports of vaccine escape, this approach provides quantitative evidence thereof. Moreover, the consistency of the data suggests the existence of some common antigenic regions. Affinity data provide a direct comparison of vaccine-induced humoral immune responses and may assist in designing or updating vaccines against recurrent viruses.

## Supplementary information

Supplementary Information

Description of Additional Supplementary Files

Supplementary Data 1

Supplementary Data 2

Supplementary Data 3

Supplementary Data 4

Reporting Summary

## Data Availability

Data generated or used in this study are available from the corresponding author upon reasonable request. Source data to generate Figs. 1–3 is available in Supplementary Data 1. Source data for Supplementary Fig. 1a–c is available in Supplementary Data 2. Source data for Supplementary Fig. 2a,b is available in Supplementary Data 3. Source data for Supplementary Fig. 3a–c is available in Supplementary Data 4.
