# Supplementary material for: Affinity of anti-spike antibodies to three major SARS-CoV-2 variants in recipients of three major vaccines
Source: Commun Med (Lond). 2022 Aug 25;2:109. doi: 10.1038/s43856-022-00174-9 (PMC9403978; doi:10.1038/s43856-022-00174-9)
Supplement: Supplementary file 1 — Supplementary Information [file 43856_2022_174_MOESM1_ESM.pdf]

Supplementary Information

Affinity of Anti-Spike Antibodies to Three Major SARS-CoV-2 Variants in Recipients of Three Major Vaccines

Patrick J. Macdonald[1]†, Jeffrey M. Schaub[1]†, Qiaoqiao Ruan[1], Carroll L. Williams[1], John C. Prostko[1], and Sergey Y. Tetin[1]*

[1]Applied Research and Technology, Abbott Diagnostics Division, Abbott Laboratories, Abbott Park, IL, USA

† P.M. and J.S. contributed equally to this work.

* Corresponding author: Sergey Y. Tetin
Address: AP-20, Abbott Laboratories, 100 Abbott Park Road, Abbott Park, IL 60064-6016.
Tel: 224-668-4661; Fax: 224-668-6498

Email: sergey.tetin@abbott.com

Table S1 – Pfizer vaccine panel: sample information and anti-RBD variant affinities. Full binding curve plots for all three vaccine panels are shown in Supplementary Fig. S1-S3. The table below contains the apparent affinities recovered from the fits for each patient sample, along with the vaccine type received, and the number of days post the initial vaccine dose that the sample was drawn. A lower bound for affinity was set at 0.0002 (see Methods).

Vaccine	Days post 1st vaccine dose	Sample ID	Affinity to WT [$\times 10^9$ M^{-1}]	Affinity to Delta [$\times 10^9$ M^{-1}]	Affinity to Omicron [$\times 10^9$ M^{-1}]
Pfizer panel 1	35	K01	3.3	0.43	0.0033
	35	K02	0.5	0.11	0.0003
	35	K03	6.7	1.11	≤0.0002
	35	K04	0.8	0.22	0.0006
	35	K05	0.8	0.21	0.0003
	35	K06	0.8	0.09	0.0005
	35	K07	1.3	0.26	0.0010
	35	K08	16.7	1.43	0.0200
	35	K09	16.7	2.00	0.0100
	35	K10	5.9	1.67	0.0050
	35	K11	7.7	1.25	0.0008
	36	K13	11.5	2.78	0.0004
	35	K14	2.3	0.33	0.0003
	35	K15	4.2	0.33	0.0010
Pfizer panel 2	53	P220	0.7	0.01	≤0.0002
	52	P222	7.1	0.20	0.0013
	53	P225	5.3	1.49	0.0004
	77	P230	1.1	0.17	0.0040
	77	P377	0.9	0.13	0.0008
	53	P378	1.1	0.05	0.0005
	52	P380	2.4	0.45	0.0003
	55	P381	1.3	0.03	0.0004
	55	P382	0.3	0.06	≤0.0002
	50	P474	1.2	0.13	≤0.0002
	52	P479	1.1	0.05	0.0004
	52	P489	5.1	2.44	0.0050

Table S2 – J&J vaccine panel: sample information and anti-RBD variant affinities.

Vaccine	Days post 1st vaccine dose	Sample ID	Affinity to WT [$\times 10^9$ M^{-1}]	Affinity to Delta [$\times 10^9$ M^{-1}]	Affinity to Omicron [$\times 10^9$ M^{-1}]
Johnson & Johnson panel 1	62	JJ001	4.0	0.40	0.0020
	61	JJ002	2.5	0.04	0.0004
	59	JJ003	0.1	0.08	≤0.0002
	60	JJ004	3.3	0.20	≤0.0002
	60	JJ005	2.0	0.20	≤0.0002
	60	JJ006	0.2	0.03	≤0.0002
	60	JJ007	0.8	0.02	≤0.0002
	61	JJ008	1.5	0.03	≤0.0002
	60	JJ009	5.3	0.59	0.0003
	60	JJ010	14.3	1.00	0.0007
	60	JJ011	2.8	0.17	0.0003
	60	JJ012	0.7	0.08	0.0033
	59	JJ013	4.5	0.25	≤0.0002
	60	JJ015	10.0	0.59	≤0.0002
J & J panel 2	35	JJ221	10.0	1.03	0.0005
	29	JJ382	1.1	0.08	≤0.0002
	29	JJ889	0.7	0.00	≤0.0002
	33	JJ897	5.6	7.14	0.0003
	45	JJ903	0.8	0.03	0.0005

Table S3 – Moderna vaccine panel: sample information and anti-RBD variant affinities.

Vaccine	Days post 1st vaccine dose	Sample ID	Affinity to WT [$\times 10^9$ M^{-1}]	Affinity to Delta [$\times 10^9$ M^{-1}]	Affinity to Omicron [$\times 10^9$ M^{-1}]
Moderna panel 1	42	G03	0.9	0.17	0.0014
	41	G04	2.0	0.21	0.0006
	42	G05	1.1	0.07	0.0010
	47	G06	0.9	0.63	0.0003
	42	G07	5.9	0.56	0.0100
	42	G08	2.3	0.11	0.0006
	44	G09	3.0	0.45	0.0025
	43	G11	1.6	0.27	0.0013
	40	G13	2.4	0.06	≤0.0002
	42	G15	3.8	0.14	≤0.0002
	42	G16	4.0	0.38	0.0007
	41	G37	1.3	0.14	≤0.0002
	44	G39	1.8	0.20	0.0005
	40	G74	8.3	2.27	0.0050
	45	G83	1.6	0.40	0.0006
Moderna panel 2	43	J10	1.7	0.40	0.0006
	41	J12	0.7	0.04	≤0.0002
	42	J14	1.4	0.04	0.0040
	43	J36	1.7	0.53	≤0.0002
	45	J40	0.5	0.05	0.0005
	42	J42	3.7	1.43	0.0010
	41	J43	8.3	0.50	0.0005
	40	J44	1.0	0.06	≤0.0002
	41	J45	2.5	0.42	≤0.0002
	42	J72	20.0	1.43	≤0.0002
	43	J93	2.9	0.17	0.0013
	43	J94	0.6	0.08	≤0.0002
	43	J95	1.7	0.20	≤0.0002
	41	J97	3.2	0.36	0.0003

Table S4 – Pfizer vaccine panel: additional sample information and anti-RBD IgG levels. Supplementary Tables. S1-S3 contain the measured apparent affinities. The table below contains the additional background information for each patient sample, along with the anti-RBD IgG levels as measured by Abbott's SARS-CoV-2 IgG II Quant ARCHITECT assay. The cut-off value for detecting the presence of anti-RBD IgG is 50 AU/mL, as per assay insert. Pre-vaccine IgG levels above this value (bolded) are presumed to indicate a previous SARS-CoV-2 infection. High concentration samples cannot be quantified above 50,000 AU/mL.

Vaccine	Sample ID	Age	Gender	Pre-Vaccine Draw	1st Vaccine	2nd Vaccine	Post-Vaccine Draw-2	Pre-Vaccine IgG [AU/mL]	Post-Vaccine IgG [AU/mL]
Pfizer panel 1	K01	54	F	05-25-21	05-25-21	06-15-21	06-29-21	0	3020
	K02	50	M	06-04-21	06-04-21	06-25-21	07-09-21	1	> 50000.0
	K03	37	M	06-04-21	06-04-21	06-25-21	07-09-21	1	27348.5
	K04	29	F	06-15-21	06-15-21	07-06-21	06-20-21	**2886**	15998.3
	K05	59	F	06-04-21	06-04-21	06-25-21	07-09-21	2	3589.4
	K06	40	M	07-08-21	07-08-21	07-29-21	08-12-21	0	18567.7
	K07	46	F	07-19-21	07-19-21	08-09-21	08-23-21	0	1259.7
	K08	37	F	07-27-21	07-27-21	08-17-21	08-31-21	**538**	45496.9
	K09	43	M	07-08-21	07-08-21	07-29-21	08-12-21	**896**	> 50000.0
	K10	51	M	07-27-21	07-27-21	08-17-21	08-31-21	**55**	35349.6
	K11	50	F	06-25-21	06-25-21	07-16-21	07-30-21	1	> 50000.0
	K13	33	F	07-24-21	07-24-21	08-15-21	08-29-21	**90**	28895.9
	K14	58	M	08-20-21	08-20-21	09-10-21	09-24-21	1	9327.7
	K15	67	F	08-06-21	08-06-21	08-27-21	09-10-21	**223**	24939.1
Pfizer panel 2	P220	63	M	03-25-21	03-25-21	04-09-21	05-17-21	0	29638
	P222	56	F	03-30-21	03-30-21	04-16-21	05-21-21	**79**	15448.3
	P225	26	F	03-26-21	03-26-21	04-12-21	05-18-21	**174**	25446
	P230	64	F	02-08-21	02-08-21	03-16-21	04-26-21	0	168.2
	P377	35	F	03-04-21	03-04-21	04-09-21	05-20-21	0	3351.6
	P378	32	F	03-05-21	03-05-21	03-23-21	04-27-21	0	13493.3
	P380	58	M	03-08-21	03-08-21	03-22-21	04-29-21	0	19250.8
	P381	38	F	03-10-21	03-10-21	03-31-21	05-04-21	3	10289.9
	P382	42	M	03-10-21	03-10-21	03-31-21	05-04-21	2	89.3
	P474	46	F	03-10-21	03-10-21	03-25-21	04-29-21	1	10590.8
	P479	23	F	03-12-21	03-12-21	03-29-21	05-03-21	2	47547.6
	P489	26	M	03-19-21	03-19-21	04-06-21	05-10-21	**116**	17601.3

Table S5 – J&J vaccine panel: additional sample information and anti-RBD IgG levels.
Pre-vaccine IgG levels above 50 (bolded values) are presumed to indicate a previous SARS-CoV-2 infection. Note: the JJ009 sample pre-vaccine IgG level at 45 is technically under the cutoff, but since it is a border case and is clearly distinct from the ~0-10 values of the other negative samples, we included it as a presumed pre-infected.

Vaccine	Sample ID	Age	Gender	Pre-Vaccine Draw	1st Vaccine	2nd Vaccine	Post-Vaccine Draw-2	Pre-Vaccine IgG [AU/mL]	Post-Vaccine IgG [AU/mL]
Johnson & Johnson panel 1	JJ001	41	F	03-07-21	03-07-21	N/A	05-08-21	2	1423
	JJ002	34	F	03-07-21	03-07-21	N/A	05-07-21	3	4178
	JJ003	40	M	03-12-21	03-12-21	N/A	05-10-21	0	155
	JJ004	64	M	03-18-21	03-18-21	N/A	05-17-21	13	305
	JJ005	50	F	04-01-21	04-01-21	N/A	05-31-21	2	233
	JJ006	61	M	04-12-21	04-12-21	N/A	06-11-21	0	92
	JJ007	31	F	04-12-21	04-12-21	N/A	06-11-21	7	212
	JJ008	44	M	05-16-21	05-16-21	N/A	07-16-21	4	817
	JJ009	40	F	07-09-21	07-09-21	N/A	09-07-21	**45**	4261
	JJ010	55	F	07-06-21	07-06-21	N/A	09-04-21	**87**	3214
	JJ011	62	M	07-06-21	07-06-21	N/A	09-04-21	**285**	8560
	JJ012	30	F	07-09-21	07-09-21	N/A	09-07-21	0	9026
	JJ013	61	M	07-27-21	07-27-21	N/A	09-24-21	1	667
	JJ015	40	M	07-09-21	07-09-21	N/A	09-07-21	6	3766
J & J panel 2	JJ221	71	F	03-25-21	03-25-21	N/A	04-29-21	**21842**	2487.6
	JJ382	31	F	04-08-21	04-08-21	N/A	05-07-21	1	325.5
	JJ889	65	F	04-28-21	04-28-21	N/A	05-27-21	5	526.4
	JJ897	49	M	05-06-21	05-06-21	N/A	06-08-21	**1064**	> 50000.0
	JJ903	61	F	05-20-21	05-20-21	N/A	06-24-21	0	666.7

Table S6 – Moderna vaccine panel: additional sample information and anti-RBD IgG levels. Pre-vaccine IgG levels above 50 (bolded values) are presumed to indicate a previous SARS-CoV-2 infection.

Vaccine	Sample ID	Age	Gender	Pre-Vaccine Draw	1st Vaccine	2nd Vaccine	Post-Vaccine Draw-2	Pre-Vaccine IgG [AU/mL]	Post-Vaccine IgG [AU/mL]
Moderna panel 1	G03	22	M	12-22-20	12-22-20	01-19-21	02-02-21	1	21964.2
	G04	26	M	12-22-20	12-28-20	01-24-21	02-07-21	3	37382.2
	G05	73	F	12-22-20	12-27-20	01-24-21	02-07-21	4	4961.4
	G06	76	M	12-22-20	12-22-20	01-20-21	02-04-21	10	3014.2
	G07	21	F	12-22-20	12-28-20	01-25-21	02-08-21	**423**	> 50000.0
	G08	45	F	12-23-20	12-23-20	01-20-21	02-03-21	0	45747.5
	G09	48	F	12-23-20	12-23-20	01-20-21	02-05-21	4	6729
	G11	56	F	12-23-20	12-23-20	01-20-21	02-04-21	1	> 50000.0
	G13	38	F	12-23-20	01-02-21	01-28-21	02-11-21	2	12381.4
	G15	39	F	12-23-20	12-23-20	01-20-21	02-03-21	**104**	23746.9
	G16	44	M	12-23-20	12-23-20	01-20-21	02-03-21	4	18794.2
	G37	33	F	01-16-21	01-23-21	02-14-21	03-01-21	1	6929.3
	G39	31	F	12-23-20	12-23-20	01-21-21	02-05-21	0	> 50000.0
	G74	47	M	01-06-21	01-06-21	02-01-21	02-15-21	5	> 50000.0
Moderna panel 2	J10	27	F	12-23-20	12-23-20	01-20-21	02-04-21	0	43833.6
	J12	75	F	12-23-20	12-23-20	01-20-21	02-02-21	0	36669.7
	J14	29	M	12-23-20	12-23-20	01-20-21	02-03-21	2	19826.3
	J36	53	F	01-07-21	01-07-21	02-05-21	02-19-21	9	15170
	J40	74	M	12-23-20	12-23-20	01-21-21	02-06-21	1	1391.7
	J42	69	F	02-07-21	02-07-21	03-07-21	03-21-21	**2570**	> 50000.0
	J43	65	F	02-07-21	02-09-21	03-08-21	03-22-21	**1043**	> 50000.0
	J44	66	F	02-07-21	02-09-21	03-08-21	03-21-21	0	14410.6
	J45	62	F	02-03-21	02-10-21	03-09-21	03-23-21	0	24098.8
	J72	73	F	02-07-21	02-09-21	03-09-21	03-23-21	**284**	37886.7
	J93	47	M	01-21-21	01-21-21	02-19-21	03-05-21	**58**	9570.9
	J94	56	F	01-21-21	01-21-21	02-19-21	03-05-21	0	30774.9
	J95	50	M	01-27-21	01-27-21	02-24-21	03-11-21	8	11960.6
	J96	49	M	01-27-21	01-27-21	02-24-21	03-11-21	0	18668.5
	J97	60	F	02-06-21	02-06-21	03-05-21	03-19-21	0	> 50000.0

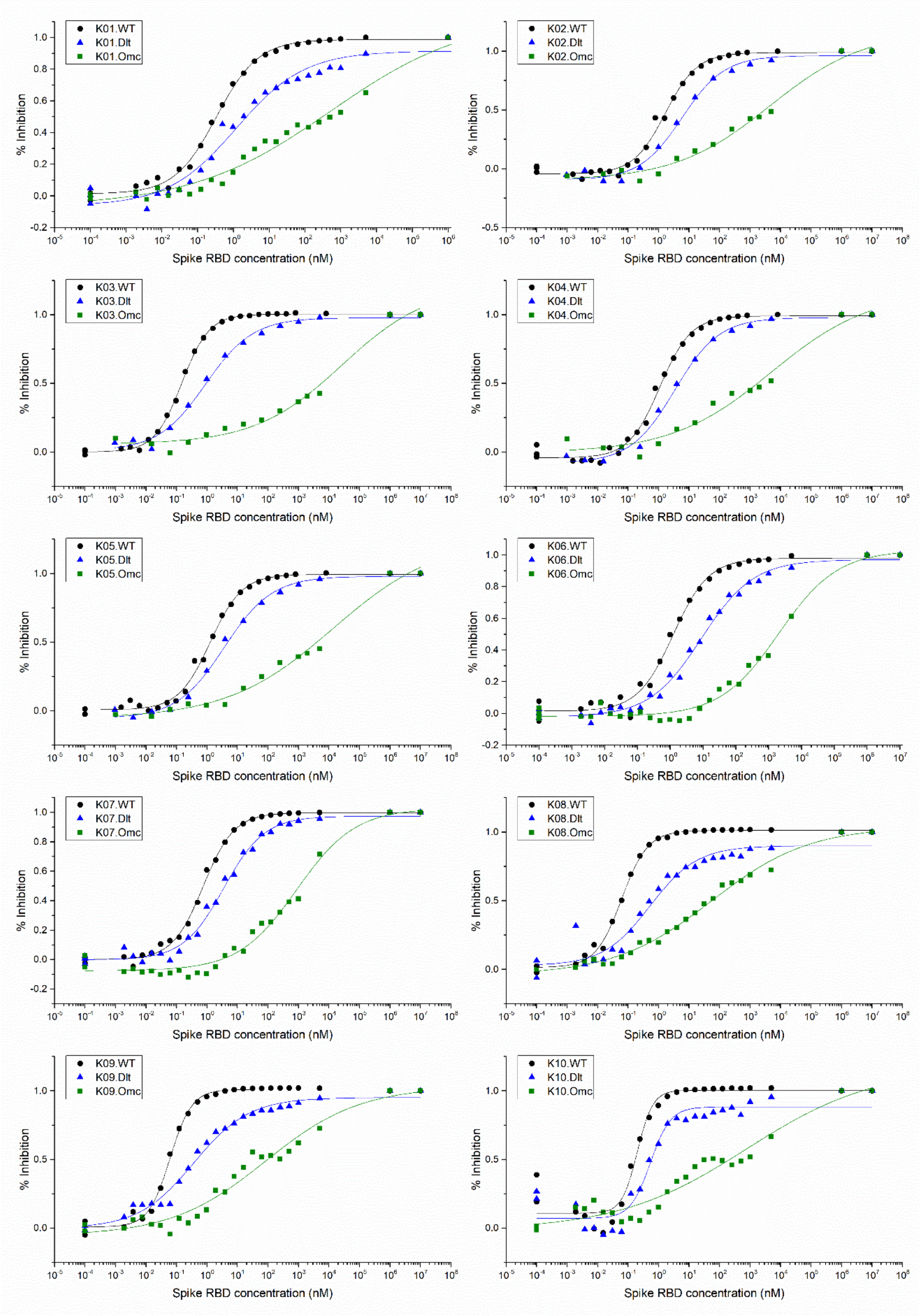

Supplementary Fig. S1a. All binding curves for Pfizer vaccine panels, Part 1.

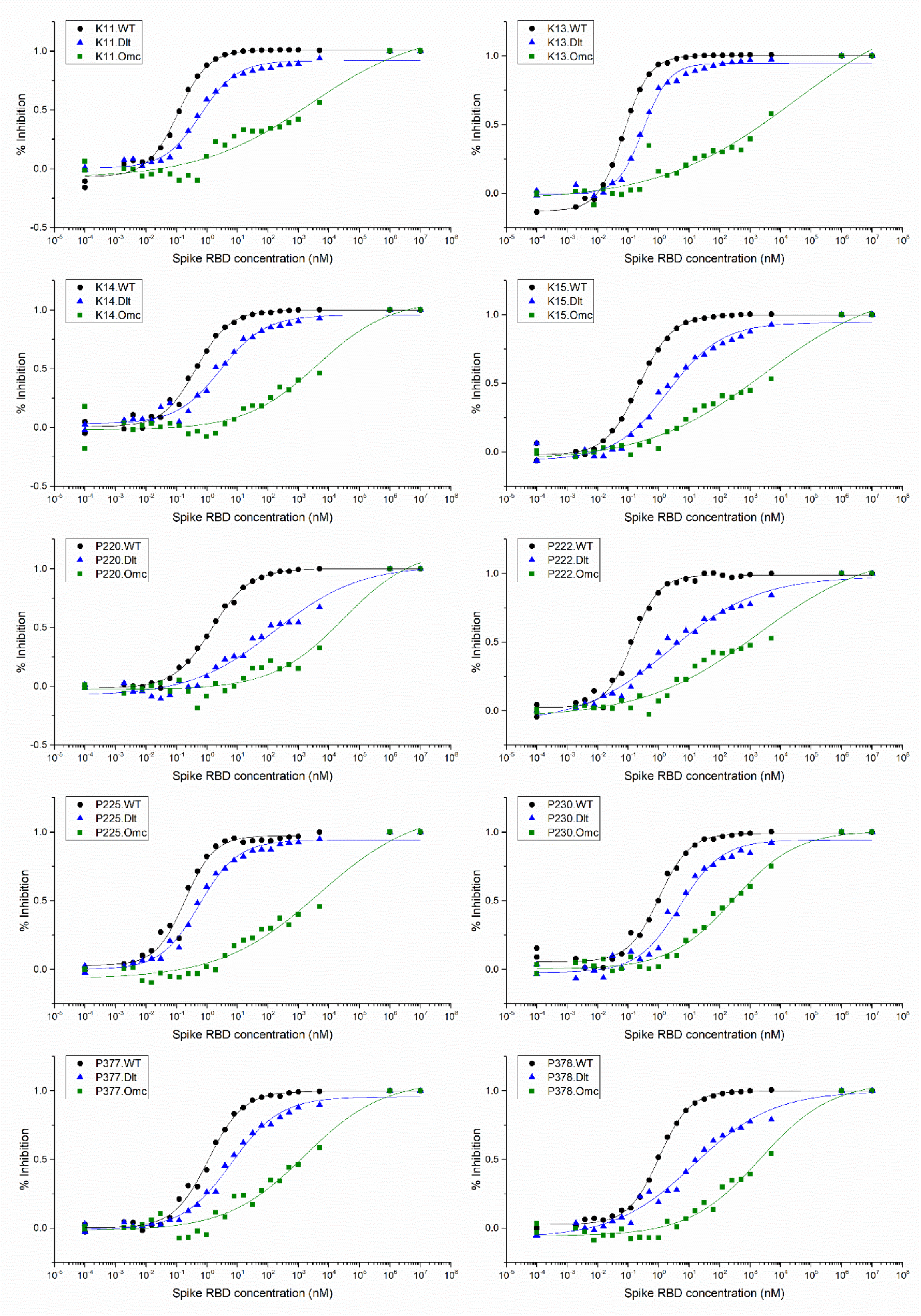

Supplementary Fig. S1b. All binding curves for Pfizer vaccine panels, Part 2.

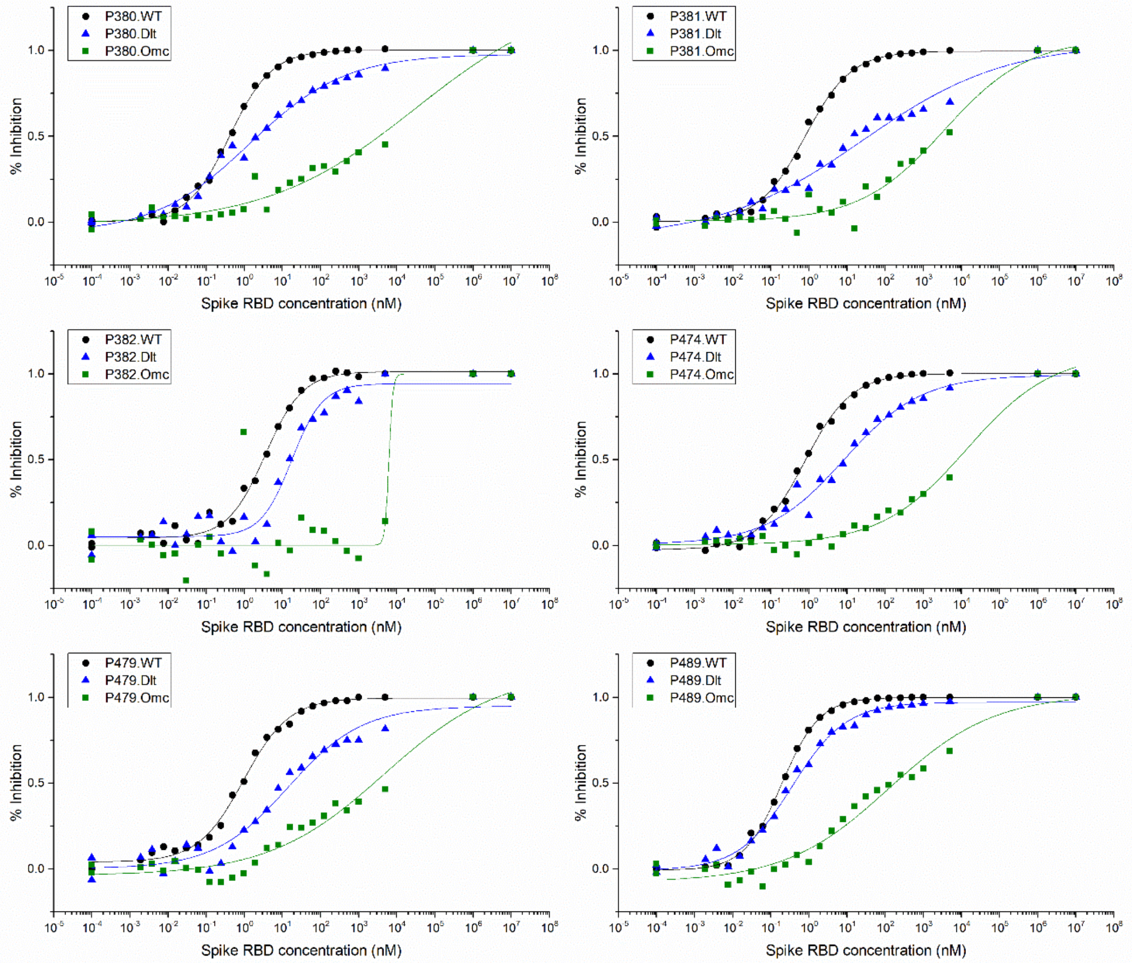

Supplementary Fig. S1c. All binding curves for Pfizer vaccine panels, Part 3.

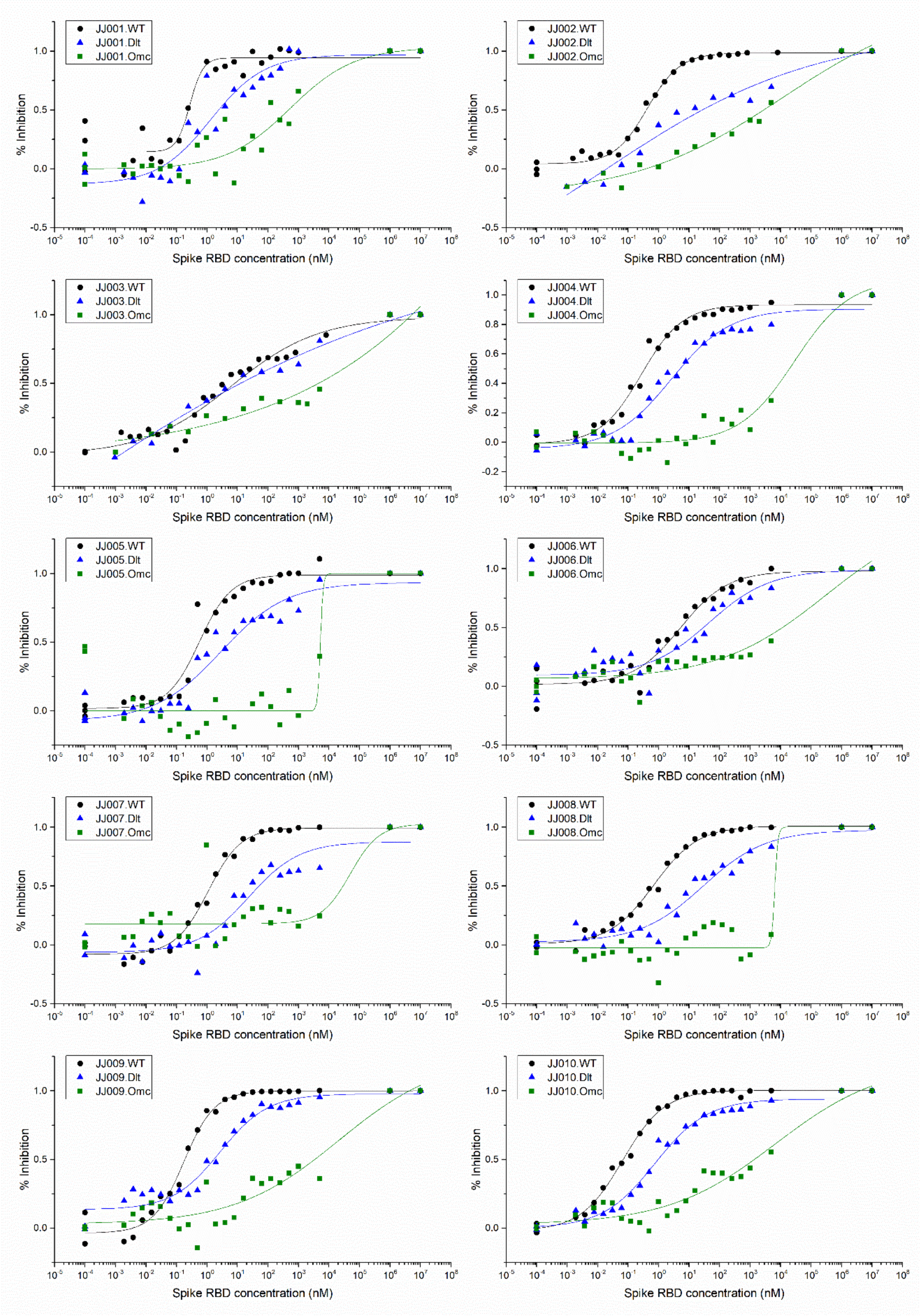

Supplementary Fig. S2a. All binding curves for Johnson & Johnson vaccine panels, Part 1.

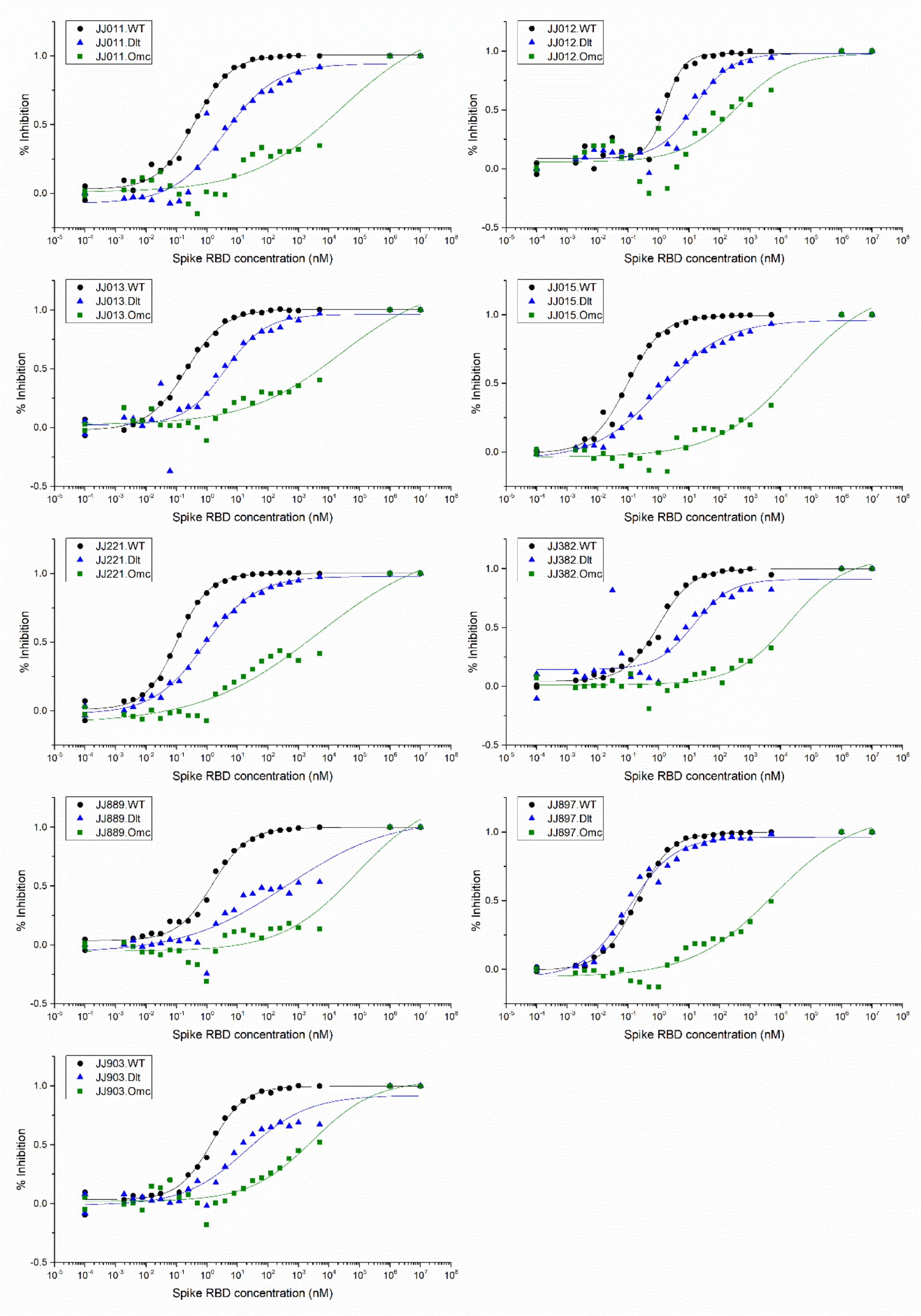

Supplementary Fig. S2b. All binding curves for Johnson & Johnson vaccine panels, Part 2.

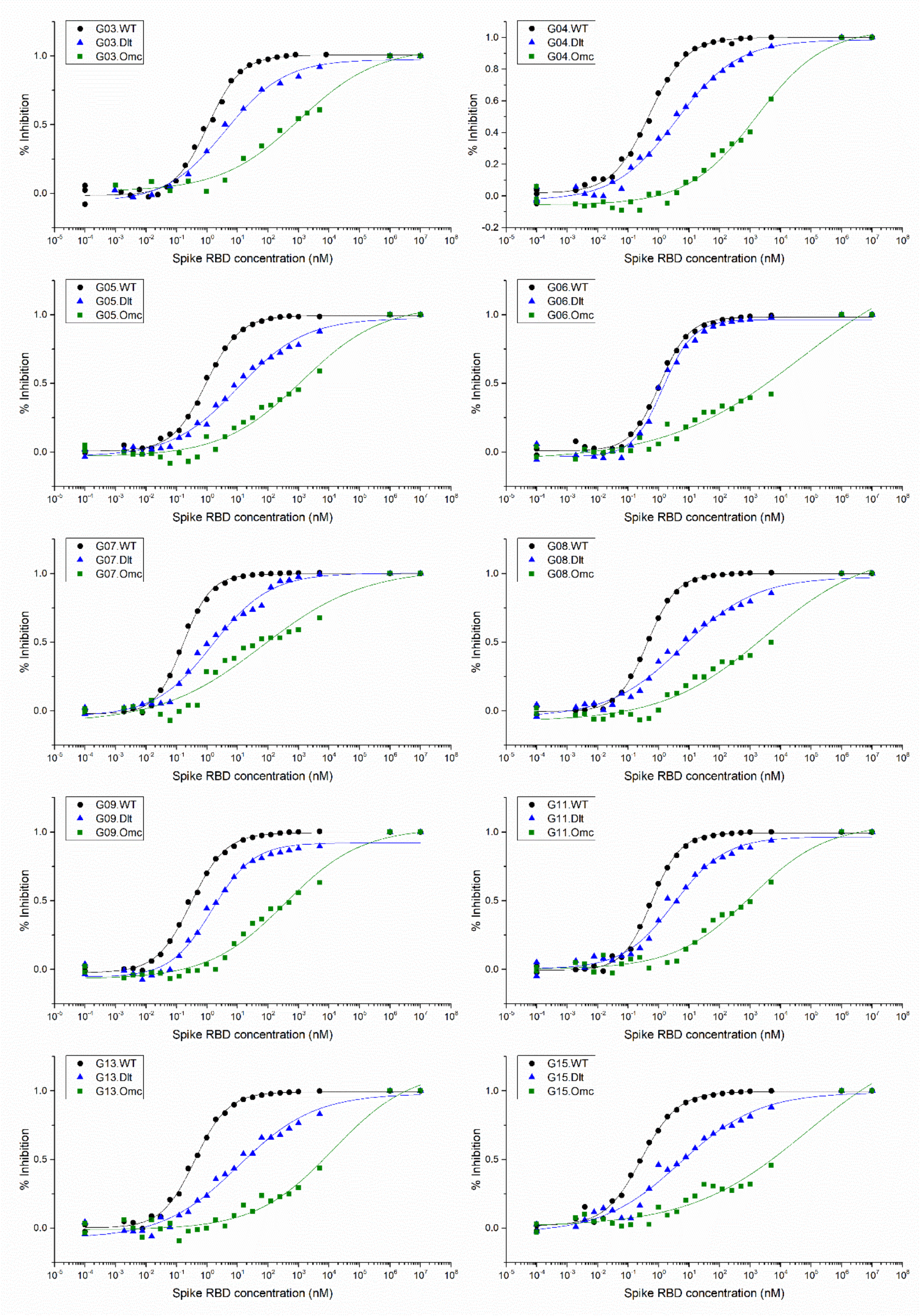

Supplementary Fig. S3a. All binding curves for Moderna vaccine panels, Part 1.

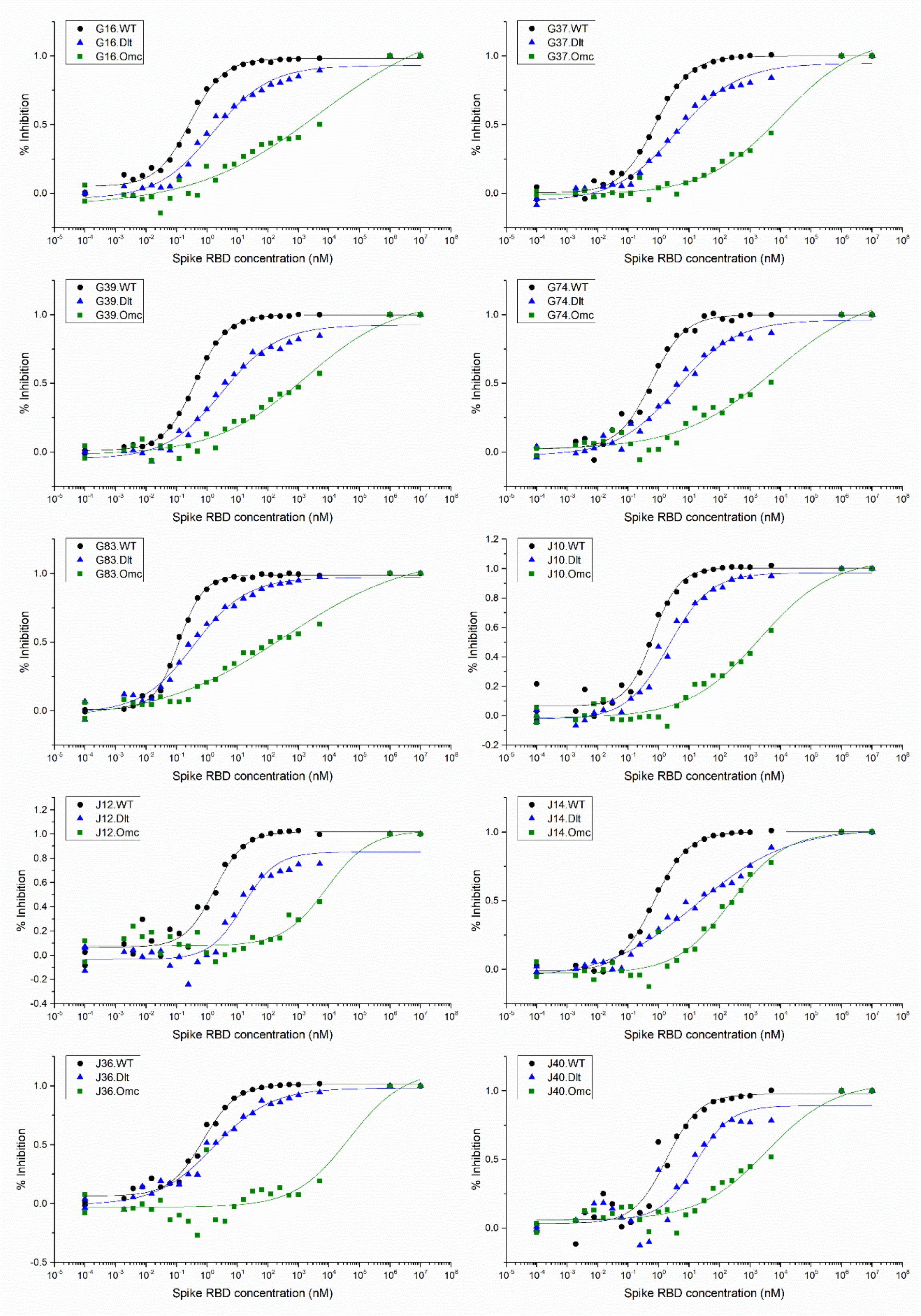

Supplementary Fig. S3b. All binding curves for Moderna vaccine panels, Part 2.

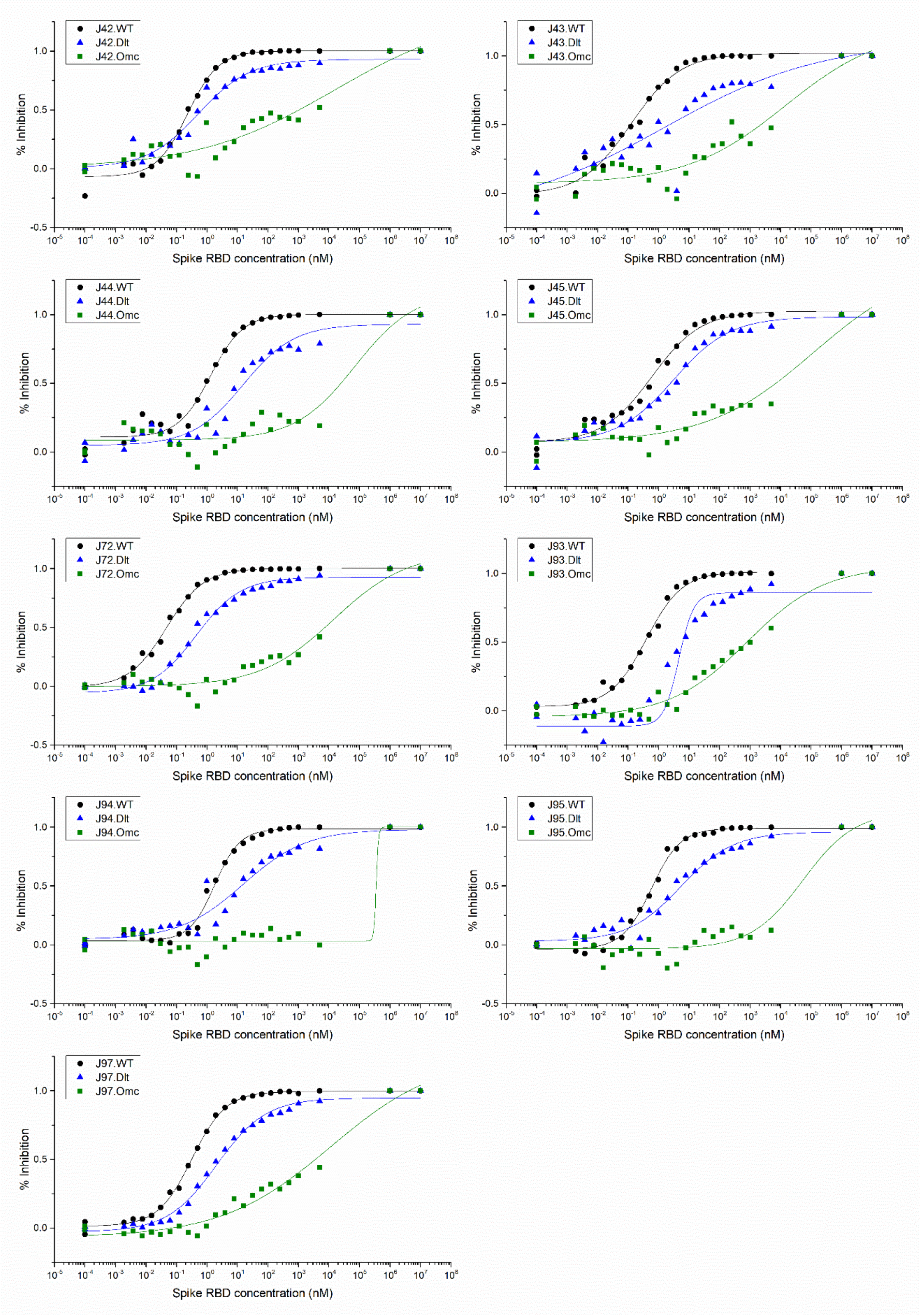

Supplementary Fig. S3c. All binding curves for Moderna vaccine panels, Part 3.

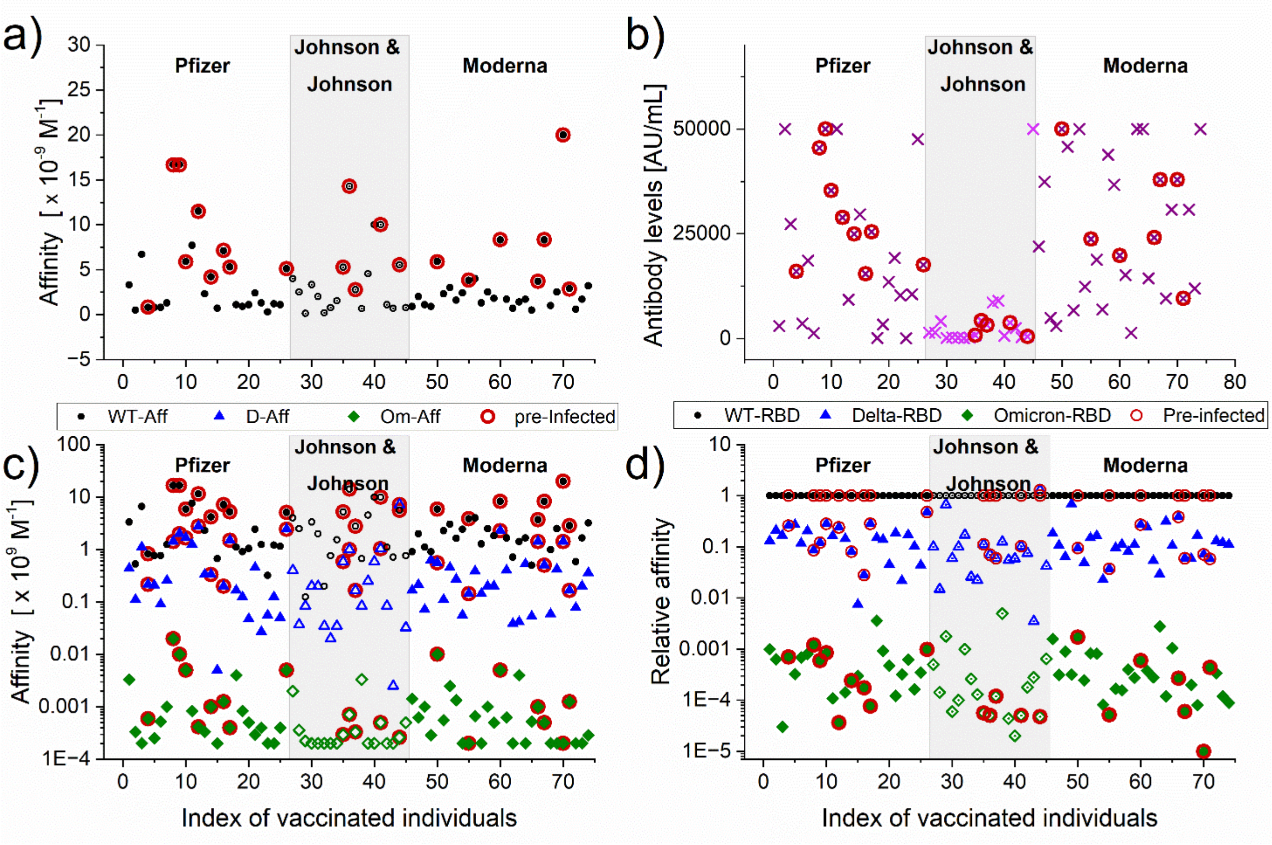

Supplementary Fig. S4 – Antibody affinities in individuals infected with SARS-CoV-2 prior to vaccination. Based on significant levels of anti-SARS-CoV-2 antibody in blood samples prior to vaccination, some individuals were identified as likely having had a WT-SARS-CoV-2 infection before they were vaccinated. a) This leads to tighter affinities toward WT-RBD, compared to the general population of vaccinated individuals, which is consistent with observations from our previous work.[1] b) In general, these individuals also showed higher antibody *concentrations*, measured by the IgG II Quant assay, which is not surprising as these individuals an additional antigen exposure compared to the rest. c) In terms of *absolute affinity* values toward Delta- and Omicron-RBD, these individuals tend toward higher affinities. c) However, the relative affinity decreases toward the variants (~10- and 1000-fold) remain consistent. Therefore, the affinity decreases for a given patient antibody toward the variants is unaffected by pre-infection status, but a stronger than average antibody toward WT-RBD is likely to have a stronger than average affinity toward Delta- and Omicron-RBD.

References

1. Macdonald PJ, Ruan Q, Grieshaber JL, et al. Affinity of anti-spike antibodies in SARS-CoV-2 patient plasma and its effect on COVID-19 antibody assays. *EBioMedicine* 2022;75:103796.
