# Supplementary material for: Affinity of anti-spike antibodies to three major SARS-CoV-2 variants in recipients of three major vaccines
Source: Commun Med (Lond). 2022 Aug 25;2:109. doi: 10.1038/s43856-022-00174-9 (PMC9403978; doi:10.1038/s43856-022-00174-9)
Supplement: Supplementary file 2 — Description of Additional Supplementary Files [file 43856_2022_174_MOESM2_ESM.pdf]

## Description of Additional Supplementary Files

**File Name:** Supplementary Data 1

**Description:** Source data for Figures 1-3.

**File Name:** Supplementary Data 2

**Description:** Antibody binding data for Pfizer vaccine recipients against WT, Delta, and Omicron RBDs.

**File Name:** Supplementary Data 3

**Description:** Antibody binding data for Johnson & Johnson vaccine recipients against WT, Delta, and Omicron RBDs.

**File Name:** Supplementary Data 4

**Description:** Antibody binding data for Moderna vaccine recipients against WT, Delta, and Omicron RBDs.
