# Supplementary material for: Affinity of anti-spike antibodies to three major SARS-CoV-2 variants in recipients of three major vaccines
Source: Commun Med (Lond). 2022 Aug 25;2:109. doi: 10.1038/s43856-022-00174-9 (PMC9403978; doi:10.1038/s43856-022-00174-9)
Supplement: Supplementary file 7 — Reporting Summary [file 43856_2022_174_MOESM7_ESM.pdf]

nature research

Corresponding author(s): S.Y.Tetin COMMSMED-22-0078-T

Last updated by author(s): Mar 28, 2022

Reporting Summary

Nature Research wishes to improve the reproducibility of the work that we publish. This form provides structure for consistency and transparency in reporting. For further information on Nature Research policies, see our Editorial Policies and the Editorial Policy Checklist.

Statistics

For all statistical analyses, confirm that the following items are present in the figure legend, table legend, main text, or Methods section.

n/a	Confirmed	
☐	☒	The exact sample size (*n*) for each experimental group/condition, given as a discrete number and unit of measurement
☐	☒	A statement on whether measurements were taken from distinct samples or whether the same sample was measured repeatedly
☐	☒	The statistical test(s) used AND whether they are one- or two-sided<br>*Only common tests should be described solely by name; describe more complex techniques in the Methods section.*
☒	☐	A description of all covariates tested
☐	☒	A description of any assumptions or corrections, such as tests of normality and adjustment for multiple comparisons
☐	☒	A full description of the statistical parameters including central tendency (e.g. means) or other basic estimates (e.g. regression coefficient) AND variation (e.g. standard deviation) or associated estimates of uncertainty (e.g. confidence intervals)
☒	☐	For null hypothesis testing, the test statistic (e.g. *F*, *t*, *r*) with confidence intervals, effect sizes, degrees of freedom and *P* value noted<br>*Give P values as exact values whenever suitable.*
☒	☐	For Bayesian analysis, information on the choice of priors and Markov chain Monte Carlo settings
☒	☐	For hierarchical and complex designs, identification of the appropriate level for tests and full reporting of outcomes
☒	☐	Estimates of effect sizes (e.g. Cohen's *d*, Pearson's *r*), indicating how they were calculated

Our web collection on statistics for biologists contains articles on many of the points above.

Software and code

Policy information about availability of computer code

Data collection	Metamorph Advanced 7.7.11.0
Data analysis	Origin 2016; JMP 16.0

For manuscripts utilizing custom algorithms or software that are central to the research but not yet described in published literature, software must be made available to editors and reviewers. We strongly encourage code deposition in a community repository (e.g. GitHub). See the Nature Research guidelines for submitting code & software for further information.

Data

Policy information about availability of data

All manuscripts must include a data availability statement. This statement should provide the following information, where applicable:

- Accession codes, unique identifiers, or web links for publicly available datasets
- A list of figures that have associated raw data
- A description of any restrictions on data availability

All data used are provided in the MS and Supplementary Info. Raw microparticle images are available upon reasonable request.
Source data to generate Figures 1-3 is available in Supplementary Data 1. Source data for Supplementary Figures 1a-1c is available in Supplementary Data 2. Source data for Supplementary Figures 2a-2b is available in Supplementary Data 3. Source data for Supplementary Figures 3a-3c is available in Supplementary Data 4.

Field-specific reporting

Please select the one below that is the best fit for your research. If you are not sure, read the appropriate sections before making your selection.

☒ Life sciences ☐ Behavioural & social sciences ☐ Ecological, evolutionary & environmental sciences

For a reference copy of the document with all sections, see nature.com/documents/nr-reporting-summary-flat.pdf

Life sciences study design

All studies must disclose on these points even when the disclosure is negative.

Sample size	Sample size n~30 was targeted to meet the normality assumptions of central limit theorem.
Data exclusions	Six samples were entirely excluded from the study due to a very low signal and/or extremely high background
Replication	Data collection was performed using an internal sampling approach
Randomization	Randomly collected blood serum samples were acquired from commercial sources. The samples were grouped only according to the vaccine received.
Blinding	All samples were unidentified.

Reporting for specific materials, systems and methods

We require information from authors about some types of materials, experimental systems and methods used in many studies. Here, indicate whether each material, system or method listed is relevant to your study. If you are not sure if a list item applies to your research, read the appropriate section before selecting a response.

Materials & experimental systems

n/a	Involved in the study	
☐	☒	Antibodies
☒	☐	Eukaryotic cell lines
☒	☐	Palaeontology and archaeology
☒	☐	Animals and other organisms
☒	☐	Human research participants
☒	☐	Clinical data
☒	☐	Dual use research of concern

Methods

n/a	Involved in the study	
☒	☐	ChIP-seq
☒	☐	Flow cytometry
☒	☐	MRI-based neuroimaging

Antibodies

Antibodies used	Donkey anti-human IgG (Cy3-labeled) was bought from Jackson Immunoresearch, 709-165-149, lot 139754.
Validation	Target: Human<br>Host: Donkey<br>Antibody Format: Whole IgG<br>Specificity: IgG (H+L)<br>Minimal Cross Reactivity: Bovine, Chicken, Goat, Guinea Pig, Syrian Hamster, Horse, Mouse, Rabbit, Rat, Sheep Serum Proteins<br>Conjugate: Cyanine Cy™3<br>Product Category: Whole IgG Affinity-Purified Antibodies<br>Clonality: Polyclonal<br>RRID: AB_2340535<br>Based on immunoelectrophoresis and/or ELISA, the antibody reacts with whole molecule human IgG. It also reacts with the light chains of other human immunoglobulins. No antibody was detected against non-immunoglobulin serum proteins. The antibody has been tested by ELISA and/or solid-phase adsorbed to ensure minimal cross-reaction with bovine, chicken, goat, guinea pig, syrian hamster, horse, mouse, rabbit, rat and sheep serum proteins, but it may cross-react with immunoglobulins from other species.<br>Please see: https://www.jacksonimmuno.com/catalog/products/709-165-149
